# *Mesembryanthemum crystallinum* -guided green synthesis of ZnO nanoparticles with selective anticancer activity via ROS-mediated mitochondrial dysfunction

**DOI:** 10.1186/s40643-026-01107-3

**Published:** 2026-08-10

**Authors:** Sara Taha, Nahed M. M. Emam, Salem S. Salem, Mohamed Nagui T. Attia, Metwally M. Montaser

**Affiliations:** 1 Department of Zoology, Faculty of Science, Arish University, North Sinai, Egypt; 2 Botany and Microbiology Department, Faculty of Science, AL-Azhar University, Nasr City, Cairo, 11884 Egypt; 3 Department of Zoology, Faculty of Science (Boys), Al-Azhar University, Nasr City, Cairo, 11884 Egypt

**Keywords:** *Mesembryanthemum crystallinum*, Green synthesis, Zinc oxide nanoparticles, Metabolomics, Selective cytotoxicity, Mitochondrial apoptosis

## Abstract

Green synthesis of zinc oxide nanoparticles (ZnO-NPs) using *Mesembryanthemum crystallinum* L. (*M. crystallinum*), a halophytic plant adapted to saline environments of North Sinai, Egypt, offers a sustainable nanofabrication strategy with inherent therapeutic potential. High-resolution LC-ESI-QTOF-MS metabolomic profiling identified 13 major phytochemicals in the ethanolic leaf extract, dominated by citramalate (peak area 31,601), L-phenylalanine (30,377), and stress-responsive organic acids, reflecting the plant’s halophytic adaptation. These redox-active compounds templated the biosynthesis of crystalline ZnO-NPs under ambient aqueous conditions, yielding spherical to quasi-spherical nanoparticles (4.4–12.2 nm primary size; 40–150 nm aggregates) with a hexagonal wurtzite structure, as confirmed by XRD and SAED. FTIR and EDX analyses verified surface functionalization by phytochemical capping agents (polyphenols, organic acids), while SEM revealed characteristic aggregation with rough surface morphology indicative of biomolecular adsorption. Biosynthesized ZnO-NPs exhibited selective cytotoxicity against human malignant melanoma A375 cells (IC₅₀ = 100.55 ± 8.6 µg/mL) with four-fold lower toxicity toward normal human skin fibroblasts (IC₅₀ = 406.01 ± 35.2 µg/mL; selectivity index ≈ 4.0). Mechanistic investigations demonstrated that nanoparticle internalization triggered robust ROS generation (~ 3.8 × 10⁴ fluorescence units), mitochondrial membrane depolarization, and mixed apoptotic/necrotic cell death (40% total death; 25% late apoptosis, 15% necrosis). Cell cycle analysis revealed cancer-selective G2/M arrest in A375 cells (20.87% vs. 8.59% control; *p* < 0.001) versus protective G1 arrest in fibroblasts (69.14% vs. 56.01% control; *p* < 0.001). Paradoxically, qPCR showed BAX downregulation in cancer cells but upregulation in normal cells, suggesting non-transcriptional execution of apoptosis in malignancies. These findings establish *M. crystallinum* as an ecologically sustainable nanofactory whose stress-adapted metabolome directs the formation of selectively cytotoxic ZnO-NPs acting through ROS-mediated mitochondrial dysfunction, a paradigm for designing plant-guided nanotherapeutics with defined molecular mechanisms.

**Graphical abstract:**

**Figure:**
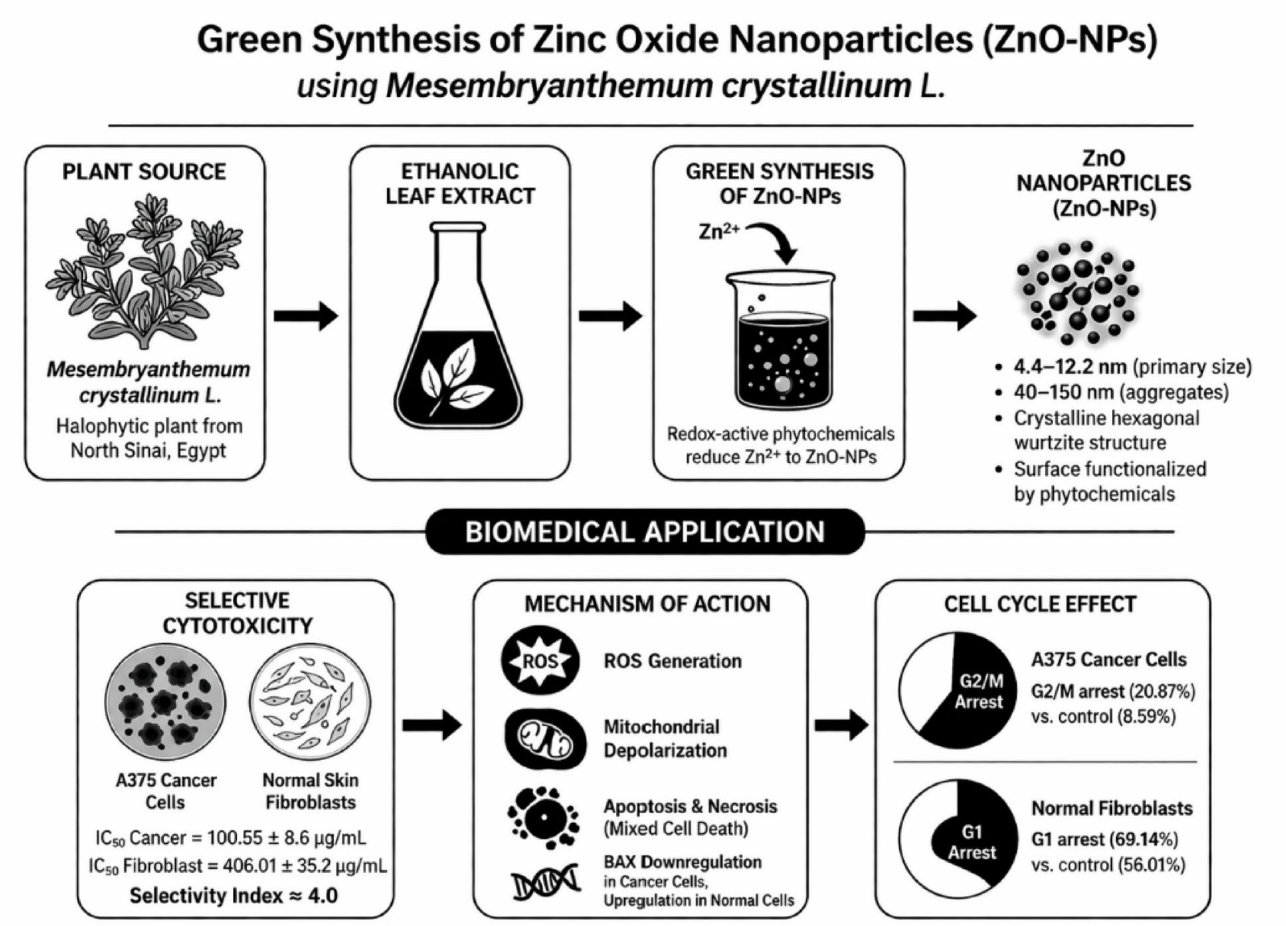

## Introduction

Cancer remains a leading cause of global mortality, with conventional therapeutic modalities frequently limited by systemic toxicity, lack of tumor specificity, and the emergence of multidrug resistance (Zafar et al. 2025). These challenges have accelerated the development of nanotechnology-based platforms that offer enhanced targeting capabilities, improved pharmacokinetics, and the potential to circumvent resistance mechanisms inherent to traditional chemotherapy (Salem 2023, Deivayanai et al. 2025, Sivalingam 2026, Salem and Fouda 2021, Selim et al. 2026, Alruhaili et al. 2025). Among nanomaterials investigated for oncological applications, zinc oxide nanoparticles (ZnO-NPs) have garnered considerable attention owing to their unique physicochemical properties, biocompatibility, and demonstrated capacity to selectively induce cytotoxicity in malignant cells while sparing normal tissues (Almuhayawi et al. 2024, El-Saadony et al. 2024, Selim et al. 2025).

The anti-cancer potential of ZnO-NPs is primarily attributed to their ability to generate reactive oxygen species (ROS) within tumor microenvironments, triggering mitochondrial dysfunction, DNA damage, cell cycle arrest, and activation of intrinsic apoptotic pathways (Mohammed et al. 2025). Unlike conventional chemotherapeutics, which often fail to discriminate between proliferating cancerous and healthy cells, ZnO-NPs exploit the heightened baseline oxidative stress and altered redox homeostasis characteristic of many cancer phenotypes, thereby achieving selective toxicity (Chen et al. 2025). Furthermore, their capacity to bypass P-glycoprotein-mediated efflux mechanisms and modulate key signaling cascades, including PI3K/Akt/mTOR and NF-κB, positions ZnO-NPs as promising candidates for overcoming multidrug resistance (Mu et al. 2025).

Conventional synthesis routes for ZnO-NPs, however, typically rely on physical or chemical methods that involve high-energy inputs, toxic reducing agents, and organic solvents, which compromise biocompatibility and generate hazardous waste (Gong et al. 2026). These limitations have catalyzed interest in green synthesis approaches that harness biological entities, particularly plant extracts, as sustainable alternatives for nanoparticle fabrication (Abdullah et al. 2021, Sivalingam 2025, Soliman and Salem 2025). Plant-mediated synthesis leverages endogenous phytochemicals (polyphenols, flavonoids, alkaloids, terpenoids, and proteins) to simultaneously reduce metal ions and cap the resulting nanoparticles, yielding stable, biocompatible nanomaterials with enhanced therapeutic potential (Sivalingam 2024, Sivalingam and Pandian 2024, Sivalingam and Pandian 2025, Sivalingam 2025, Soliman and Salem 2025). This strategy aligns with green chemistry principles by eliminating toxic reagents, reducing energy consumption, and minimizing environmental footprint (Okaiyeto et al. 2024).

*Mesembryanthemum crystallinum* L. (Aizoaceae; *M. crystallinum*), commonly known as ice plant, represents an underexplored yet highly promising candidate for green nanoparticle synthesis. This halophytic succulent has evolved sophisticated physiological adaptations to thrive in saline, arid environments, including facultative Crassulacean acid metabolism (CAM) photosynthesis and specialized epidermal bladder cells that sequester sodium ions at concentrations up to 1 M (Rincón-Cervera et al. 2024). These adaptations correlate with a rich phytochemical repertoire; recent LC-QTOF/MS profiling identified 178 distinct compounds in *M. crystallinum*, with lipids and lipid-like molecules constituting 69.8% of its composition, alongside phenolic derivatives (e.g., ferulic acid, 4-hydroxy-3-methoxybenzamide), nucleosides, and amino acids (Yadav et al. 2020). The plant’s documented antioxidant capacity, mediated by enzymatic systems (superoxide dismutase, catalase, ascorbate peroxidase) and non-enzymatic scavengers (polyphenols, flavonoids), suggests inherent redox activity suitable for metal ion reduction (Maury et al. 2020, Bas 2026).

Critically, *M. crystallinum* extracts have demonstrated direct antitumor effects. Seo and Ju reported dose-dependent growth inhibition and apoptosis induction in HCT116 human colon cancer cells, with the butanol fraction exhibiting superior activity compared to crude ethanol extracts (Seo and Ju 2019). More recently, Rincón-Cervera et al. documented concentration- and time-dependent antiproliferative effects against HT-29 colorectal adenocarcinoma cells, with moderate selectivity toward malignant versus normal cells (selectivity index 1.2–1.7) (Rincón-Cervera et al. 2024). These findings implicate phenolic compounds, carotenoids, and other bioactive constituents in the plant’s cytotoxic mechanisms, likely acting synergistically to disrupt cancer cell viability.

Despite these promising attributes, a significant research gap persists: no published studies have explored *M. crystallinum* L. as a phyto-reductant for ZnO-NP biosynthesis, nor have the molecular mechanisms underlying the cytotoxic effects of such biosynthesized nanoparticles been systematically investigated. Existing literature on plant-mediated ZnO-NPs predominantly features common medicinal species (e.g., *Aloe vera*, *Ocimum basilicum*), overlooking halophytes with unique stress-adaptive metabolomes that may confer distinctive nanoparticle properties (Yahya et al. 2022). Moreover, most green-synthesized ZnO-NP studies limit assessment to basic cytotoxicity screening without probing downstream molecular events, such as mitochondrial membrane depolarization, caspase activation, Bax/Bcl-2 modulation, or ROS-mediated DNA damage, that define mechanistic anti-cancer efficacy (Wang et al. 2024).

This study, therefore, aimed to biosynthesize ZnO-NPs using *M. crystallinum* L. leaf extract via an eco-friendly green approach and conduct a comprehensive in vitro cytotoxic and molecular assessment against human cancer cell lines. We hypothesized that phytochemicals inherent to this halophyte would yield ZnO-NPs with enhanced stability and anti-cancer potency, acting through ROS-mediated mitochondrial apoptosis. Specific objectives included: physicochemical characterization of biosynthesized nanoparticles using XRD, FTIR, TEM, and SEM/EDX; evaluation of dose-dependent cytotoxicity against selected carcinoma cell lines; molecular dissection of cell death mechanisms through assessment of ROS generation, mitochondrial membrane potential disruption, and expression modulation of key apoptotic regulators (Bax and Bcl-2). By bridging green nanotechnology with halophyte phytochemistry, this work addresses an unmet need for sustainable nanotherapeutics with defined molecular mechanisms of action.

## Materials and methods

### Chemicals and reagents

All chemicals and reagents were of analytical grade or higher purity. Methanol and acetonitrile (HPLC grade), formic acid (≥ 98%), and sodium hydroxide pellets (anhydrous) were obtained from Fisher Scientific (UK) and Sigma-Aldrich (Germany). Ammonium formate (mass spectrometry grade, ≥ 98%) was purchased from Sigma-Aldrich (Germany). Ultrapure water (18.2 MΩ·cm at 25 °C) was generated using a Milli-Q water purification system (Millipore, USA). Commercial ZnO-NPs were procured from Nawah Scientific (Cairo, Egypt), and Doxorubicin was sourced from Sigma-Aldrich (Germany). Cell culture media and supplements, including Dulbecco’s Modified Eagle Medium (DMEM), fetal bovine serum (FBS), L-glutamine, and antibiotic-antimycotic solution, were acquired from SPL Life Sciences (Seoul, South Korea) and Biowest (Nuaillé, France). Potassium bromide (KBr) for FTIR analysis was obtained from Agilent (USA). All reagents were used as received without further purification.

### Plant material and extract preparation

*M. crystallinum* L. specimens were collected from Arish, North Sinai, Egypt, and authenticated at the Department of Pharmacognosy, Faculty of Pharmacy, Sinai University, where a voucher specimen was deposited. Fresh leaves were thoroughly washed with distilled water to remove surface contaminants, shade-dried at ambient temperature (25 ± 2 °C) for 7 days, and coarsely powdered using a mechanical grinder.

For metabolomic profiling, an additional extract was prepared by sonicating 10 g of dried powder twice for 3 h at 25 °C with 100 mL of 74% ethanol (v/v) using a magnetic stirrer. The ethanolic extract was filtered through 11 μm pore filter paper (Whatman, USA), completely evaporated under reduced pressure using a rotary evaporator (40 °C, 150 mbar), and stored as a dried powder at − 20 °C until LC-MS analysis. Prior to analysis, the dried extract was reconstituted in LC-MS-grade methanol at 1 mg/mL (Oh et al. 2024).

### Metabolomic profiling by LC-MS/MS

For untargeted metabolomic analysis, 50 mg of dried ethanolic extract was dissolved in 1 mL of reconstitution solvent (water: methanol: acetonitrile, 50:25:25, v/v/v). The solution was vortexed vigorously for 2 min, sonicated for 10 min, and centrifuged at 10,000 rpm for 10 min. The supernatant was diluted to a final concentration of 2.5 µg/µL, and 10 µL was injected for LC-MS analysis. A solvent blank was prepared and analyzed under identical conditions (Mohammed et al. 2021).

Chromatographic separation was performed on a Sciex ExionLC system coupled to a Sciex TripleTOF 5600 + mass spectrometer. Separation was achieved using a Waters XSelect HSS T3 C18 column (100 Å, 2.5 μm, 2.1 × 150 mm) maintained at 40 °C. The mobile phase consisted of (A) 5 mM ammonium formate buffer (pH 8.0) with 1% methanol for negative ionization mode; (B) 5 mM ammonium formate buffer (pH 3.0) with 1% methanol for positive ionization mode; and (C) 100% acetonitrile. The gradient program was as follows: 0–1 min, 95% A/B and 5% C; 1–21 min, linear gradient to 5% A/B and 95% C; 21–28 min, 5% A/B and 95% C; 28.1–35 min, re-equilibration to initial conditions. The flow rate was 0.3 mL/min, and the injection volume was 10 µL. In negative ionization mode, MS1 acquisition employed high-resolution time-of-flight scanning (mass range 50–1000 Da) with the following parameters: nebulizer gas (GS1) 45 psi, heater gas (GS2) 45 psi, curtain gas 25 psi, source temperature 500 °C, and ion spray voltage − 4500 V. MS2 data were acquired using information-dependent acquisition with a declustering potential of 80 V, collision energy of − 35 eV, and collision energy spread of 15 eV. Precursor ions with intensity > 200 counts per second triggered MS/MS fragmentation, with a maximum of 15 candidate ions per cycle and dynamic background subtraction enabled (Hegazy et al. 2021).

Raw data were processed using MS-DIAL 4.9 with MS1 and MS2 mass tolerances of 0.01 and 0.05 Da, respectively. Peak detection parameters included a minimum peak height of 1000 amplitude, a mass slice width of 0.05 Da, smoothing over 2 scans, and a minimum peak width of 5 scans. Metabolite identification was performed against the ReSpect database (1573 records) with an identification score cutoff of 70% and mass accuracy error ≤ 10 ppm. MasterView 1.1 software was used for feature extraction, with signal-to-noise ratios > 10 and sample-to-blank intensity ratios > 3.

### Green synthesis of ZnO-NPs

ZnO-NPs were synthesized using a one-pot green approach, with *M. crystallinum* aqueous extract serving as both the reducing and capping agent. Briefly, 100 mL of freshly prepared plant extract was mixed with 100 mL of 0.1 M zinc nitrate hexahydrate solution in a 500 mL Erlenmeyer flask to achieve a 1:1 volume ratio. A control mixture containing plant extract without zinc precursor was prepared under identical conditions. Both mixtures were incubated in a rotary shaker at 200 rpm and 25 °C for 24 h. Nanoparticle formation was monitored by visual observation of color change (Farooq et al. 2022).

The synthesized nanoparticles were separated by centrifugation at 9000 rpm for 20 min at 10 °C (SiGMA 2K15, Germany), washed three times with distilled water to remove unreacted precursors and biomolecules, and dried at 80 °C for 48 h. The resulting powder was stored at 4 °C in amber vials for subsequent characterization and biological assays (Al-Rajhi et al. 2022, Abdelghany et al. 2023, Salem et al. 2022).

### Physicochemical characterization of ZnO-NPs

#### Fourier Transform Infrared Spectroscopy (FTIR)

FTIR analysis was performed to identify functional groups involved in the reduction and stabilization of ZnO-NPs. Samples were prepared by mixing 1 mg of dried nanoparticles with 2.5 mg KBr and pressing into transparent pellets. Spectra were recorded on an Agilent Cary 630 FTIR spectrometer over the range 400–4000 cm⁻¹ at 4 cm⁻¹ resolution.

#### X-Ray Diffraction (XRD) analysis

Crystalline structure and phase purity were determined by XRD using a Shimadzu XRD-6000 diffractometer equipped with Cu-Kα radiation (λ = 1.5406 Å) and a nickel filter. Patterns were recorded over 2θ = 10–80° at 40 kV and 30 mA with a scan rate of 2°/min. Crystallite size was calculated using the Debye-Scherrer equation: D = kλ/(β cos θ), where D is crystallite size (nm), k is the shape factor (0.9), λ is X-ray wavelength, β is full width at half maximum (FWHM) in radians, and θ is the Bragg angle.

#### Scanning Electron Microscopy (SEM) with Energy-Dispersive X-Ray (EXD) spectroscopy

Morphology and elemental composition were examined using a field-emission scanning electron microscope (QUANTA FEG250) operated at 20 kV. Samples were mounted on aluminum stubs, sputter-coated with gold (10 nm thickness), and imaged at multiple magnifications. Simultaneous EDX analysis was performed to confirm elemental composition and distribution.

#### Transmission Electron Microscopy (TEM)

TEM (JEOL 1010) was employed to determine nanoparticle size distribution, shape, and aggregation state. A drop of nanoparticle suspension (0.1 mg/mL in distilled water) was placed on a carbon-coated copper grid, air-dried, and examined at 80 kV accelerating voltage.

### Cell culture

Human malignant melanoma A375 cells (ATCC, USA) and primary normal human skin fibroblasts (hFB; BCRJ, Brazil) were maintained in DMEM supplemented with 10% FBS, 2 mM L-glutamine, and 1% antibiotic-antimycotic solution. Cultures were incubated at 37 °C in a humidified 5% CO₂ atmosphere and subcultured at 80% confluence using 0.25% trypsin-EDTA. For experiments, cells were seeded at appropriate densities and allowed to adhere overnight before treatment.

### Experimental design and treatment groups

Five experimental groups were established to comprehensively evaluate biological effects: (1) Plant extract group: Cells treated with *M. crystallinum* ethanolic extract at ½ IC₅₀ concentration to assess inherent phytochemical activity. (2) Commercial ZnO-NP group: Cells exposed to commercially available ZnO-NPs (Nawah Scientific) at ½ IC₅₀ to provide a benchmark for synthesized nanoparticles. (3) Synthesized ZnO-NP group: Cells treated with green-synthesized ZnO-NPs at ½ IC₅₀ (primary experimental group). (4) Doxorubicin control group: Cells treated with Doxorubicin (3.125–100 µg/mL for hFB; 0.312–10 µg/mL for A375) as a positive control for cytotoxicity and mechanistic studies. (5) Untreated control group: Cells maintained in culture medium with equivalent vehicle volumes to establish baseline parameters.

All treatments were administered in triplicate across three independent experimental replicates. Stock solutions of test compounds were prepared in phosphate-buffered saline (PBS) at 10 mg/mL and serially diluted in culture medium to achieve final assay concentrations.

### Biological assays

#### Cytotoxicity assessment (MTT assay)

Cytotoxicity was evaluated using the MTT reduction assay (Hansen et al. 1989). Cells were seeded at 1 × 10⁴ cells/well in 96-well plates and treated with serial dilutions of test compounds (15.625–500 µg/mL) for 48 h. Doxorubicin served as a positive control at concentrations yielding a full dose-response curve. Following treatment, 40 µL of MTT solution (5 mg/mL in 0.9% NaCl) was added to each well, and the plates were incubated for 4 h at 37 °C. Formazan crystals were dissolved in 180 µL acidified isopropanol (0.1 N HCl in isopropanol), and absorbance was measured at 570 nm using a microplate reader (FLUOstar OPTIMA, BMG LABTECH). Cell viability was calculated as:

$$ Cell\:viability\:\left( \% \right)\: = \:\:\frac{{Absorbance\:of\:treated\:cells}}{{Absorbance\:of\:control\:cells}}\: \times \:100 $$

IC₅₀ values were determined by non-linear regression analysis of dose-response curves using a five-parameter logistic equation.

#### Intracellular Reactive Oxygen Species (ROS) detection

ROS generation was quantified using 2’,7’-dichlorofluorescin diacetate (DCFH-DA). Cells seeded at 5 × 10⁴ cells/well in black 96-well plates were treated with test compounds at ½ IC₅₀ for 24 h. Cells were then incubated with DCFH-DA (10 µM) for 30 min at 37 °C in the dark, washed twice with PBS, and fluorescence intensity was measured at excitation/emission wavelengths of 500/525 nm. Tert-butyl hydroperoxide (100 µM) served as a positive control for ROS induction.

#### Mitochondrial membrane potential (ΔΨm) assessment

ΔΨm was evaluated using MitoTracker Red CMXRos, a cationic dye that accumulates in active mitochondria in a membrane potential-dependent manner (Zorova et al. 2018, Li et al. 2020). Cells seeded at 5 × 10³ cells/well in black 96-well plates or 1 × 10⁴ cells/well in 8-well chamber slides were treated with test compounds at ½ IC₅₀ for 24 h. Cells were then incubated with MitoTracker Red CMXRos (100 nM) for 45 min at 37 °C, washed three times with PBS, and fluorescence was measured at 579/599 nm (excitation/emission) using a microplate reader or visualized by fluorescence microscopy (AxioImager Z2, Zeiss).

#### Morphological assessment of cell death

Cell death modes were distinguished by differential nuclear morphology using AO/EtBr dual staining (Leite et al. 1999). Cells seeded at 2 × 10⁴ cells/well in 8-well chamber slides were treated with test compounds at ½ IC₅₀ for 24 h, stained with AO (100 µg/mL) and EtBr (100 µg/mL) for 10 min, washed with PBS, and immediately examined under fluorescence microscopy. Viable cells displayed uniform green nuclei; early apoptotic cells showed green nuclei with condensed or fragmented chromatin; late apoptotic cells exhibited orange-red condensed nuclei; and necrotic cells displayed uniformly orange-red nuclei with intact morphology.

#### Cell cycle analysis by flow cytometry

Cell cycle distribution was determined by propidium iodide (PI) staining and flow cytometry [100]. Cells (1 × 10⁶) seeded in 6-well plates were treated with test compounds at ½ IC₅₀ for 48 h, harvested by trypsinization, washed twice with PBS, fixed in 70% ethanol at 4 °C for 30 min, and treated with RNase A (100 µg/mL) for 30 min at 37 °C. Cells were then stained with PI (50 µg/mL) for 30 min in the dark and analyzed using a CytoFLEX flow cytometer (Beckman Coulter). Cell cycle phase distribution was determined using ModFit LT software based on DNA content histograms.

#### Quantitative real-time PCR analysis

Expression of apoptotic markers (BAX, BCL2) was evaluated by qPCR. A375 and hFB cells (1 × 10⁶ cells/well in 6-well plates) were treated with test compounds at ½ IC₅₀ for 48 h. Total RNA was extracted using the RNeasy Mini Kit (Qiagen), and concentration/purity were assessed by NanoDrop spectrophotometry (A260/A280 ratio 1.8–2.0). One microgram of RNA was reverse-transcribed using the QuantiTect Reverse Transcription Kit (Qiagen). qPCR was performed using QuantiTect Primer Assays (Qiagen) and QuantiNova SYBR Green PCR Kit on an Agilent Mx3000P system with the following cycling conditions: 95 °C for 5 min; 40 cycles of 95 °C for 10 s and 60 °C for 30 s. Gene expression was normalized to GAPDH and calculated using the 2^−ΔΔCt^ method.

### Statistical analysis

All experiments were performed in triplicate across three independent biological replicates. Data are expressed as mean ± standard deviation (SD). Dose-response curves were generated by non-linear regression analysis using a five-parameter logistic equation. Statistical significance among treatment groups was determined by one-way analysis of variance (ANOVA) followed by Tukey’s multiple comparison test at α = 0.05. All analyses were conducted using GraphPad Prism v10.4.1 (GraphPad Software, USA).

## Results and discussion

### Phytochemical profiling of *M. crystallinum* L. extract reveals a stress-adapted metabolome rich in redox-active compounds

**Fig. 1:**
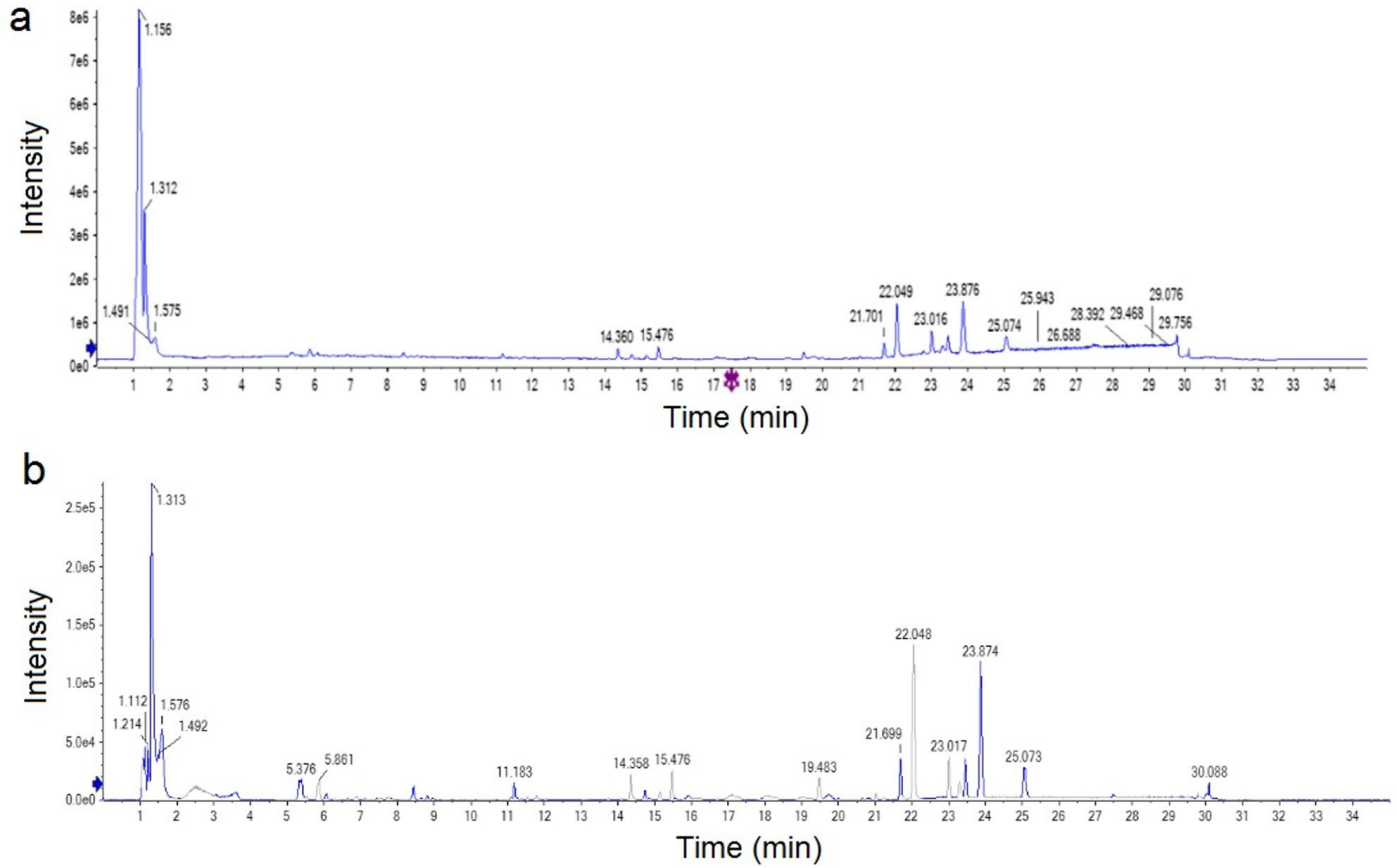
High-resolution LC-MS chromatographic profiles of *M. crystallinum* leaf extract in negative ionization mode. **a** Total ion chromatogram (TIC) acquired over a 35-min gradient elution on a Waters XSelect HSS T3 C18 column, showing temporal distribution of ionizable metabolites with major peaks at 1.156, 1.312, 14.360, 15.476, 21.701, 22.049, 23.016, 23.876, 25.074, 28.076, and 29.756 min. **b** Base peak chromatogram (BPC) highlighting the most abundant ions at each retention time, with the dominant feature at 1.313 min (intensity ~ 2.95 × 10³ arbitrary units) followed by secondary peaks at 1.112, 1.214, 1.492, 1.576, 5.376, 5.861, 11.183, 14.358, 15.476, 19.483, 21.699, 22.048, 23.017, 23.874, 25.073, and 30.088 min. The chromatographic profile indicates enrichment of highly ionizable acidic metabolites, including phenolic acids, flavonoid glycosides, and organic acid derivatives. All annotated features satisfied stringent quality criteria: mass accuracy ≤ ± 10 ppm, signal-to-noise ratio > 10, and sample-to-blank intensity ratio > 3.

High-resolution LC-ESI-QTOF-MS analysis in negative ionization mode identified 13 major metabolites in the ethanolic leaf extract of *M. crystallinum*, with citramalate emerging as the most abundant compound (peak area 31,601) (Table 1; Fig. 1) (Sugimoto et al. 2021, Islamova et al. 2023). This branched-chain α-keto acid, a key intermediate in isoleucine biosynthesis, has been implicated in microbial stress adaptation but remains underexplored in higher plants. Its prominence in *M. crystallinum*, collected from the saline coastal environment of Arish, North Sinai, suggests a potential role in osmotic adjustment and carbon-nitrogen balance under salinity stress (Guan et al. 2020). This hypothesis is reinforced by the co-occurrence of multiple organic acids (citraconic, maleic, methylsuccinic, lactic), indicating active primary metabolism geared toward pH homeostasis and redox regulation in harsh environments.

L(–)-Phenylalanine (peak area 30,377), a precursor to the phenylpropanoid pathway, was detected alongside 3-(4-hydroxyphenyl) prop-2-enoic acid, a structural analog of p-coumaric acid (Yadav et al. 2020). These findings confirm the plant’s capacity to synthesize phenolic compounds with established roles in UV protection and anti-microbial defense, critical adaptations for a halophyte exposed to intense solar radiation and microbial challenges in saline habitats (Kavaliauskas et al. 2024). The dark brown coloration of the crude extract visually corroborates this high phenolic content, a trait commonly associated with halophytic stress resilience (Cebani et al. 2024).

Notably, L-5-oxoproline, a cyclic derivative of glutamate and intermediate in the γ-glutamyl cycle of glutathione metabolism, was identified with substantial abundance (peak area 15,405). Elevated oxoproline levels often correlate with oxidative stress or enhanced glutathione turnover, aligning with *M. crystallinum*’s documented capacity to accumulate antioxidants under high salinity (Singh et al. 2025). This observation reinforces the critical role of sulfur-containing metabolites in the plant’s stress defense architecture (Abooshahab et al. 2025). Similarly, trans-4-hydroxy-L-proline, enriched in hydroxyproline-rich glycoproteins such as extensins, suggests active cell wall remodeling to maintain structural integrity under osmotic stress, a recognized hallmark of halophytic adaptation (Castilleux et al. 2021).

The detection of nucleotide derivatives (uridine 5’-monophosphate, inosine 5’-diphosphate, xanthosine 5’-monophosphate) and gamma-linolenic acid, an omega-6 polyunsaturated fatty acid precursor to jasmonate signaling molecules, further illustrates a metabolome finely tuned for environmental resilience (Rinne et al. 2024, Xiao et al. 2024). While several compounds (phenylalanine, gamma-linolenic acid, hydroxycinnamic acid derivatives) have been previously reported in *M. crystallinum* (Oh et al. 2024, Wasternack and Song 2016), our study significantly expands the known metabolome by highlighting citramalate, citraconic acid, and methylsuccinic acid as major constituents. These compounds have not been emphasized in prior LC-MS analyses, suggesting that our extraction protocol (74% ethanol) and chromatographic conditions (high-pH mobile phase, pH 8) enhanced detection of stress-induced organic acids (Bergmann et al. 2024).

**Table 1:** Comparative summary of major metabolites identified in *M. crystallinum* extract by LC-MS (negative mode, present study) and previously reported LC-MS studies

Compound	Retention time (min)	m/z [M-H]-	Relative abundance (peak area)	Reported in literature	Reference
Citramalate	1.39	147.03	31,601	—	—
L-(-)-Phenylalanine	3.06	164.07	30,377	Detected	(Oh et al. 2024)
Citraconic Acid	1.22	128.98	24,948	—	—
Maleic Acid	1.07	114.99	24,557	—	—
Methylsuccinic acid	1.07	130.98	22,456	—	—
Citraconic Acid	1.32	129.02	19,937	—	—
Gamma-Linolenic acid	21.03	277.22	17,189	Detected	(Oh et al. 2024)
Uridine 5’-monophosphate	5.52	323.12	15,741	Uridine detected	(Oh et al. 2024)
L-5-Oxoproline	1.63	128.04	15,405	—	—
Inosine-5’-diphosphate	1.32	427.01	15,010	—	—
Xanthosine-5’-monophosphate	7.75	363.08	13,283	Nucleosides reported	(Oh et al. 2024)
3-(4-Hydroxyphenyl)Prop-2-Enoic Acid	6.88	163.04	7650	Hydroxycinnamic acids	(Oh et al. 2024)
trans-4-Hydroxy-L-proline	1.42	130.09	5805	—	—
Lactic acid	1.38	89.02	5383	Organic acids reported	(Oh et al. 2024)

### Green synthesis and physicochemical characterization of ZnO-NPs

#### Morphology and elemental composition

**Fig. 2:**
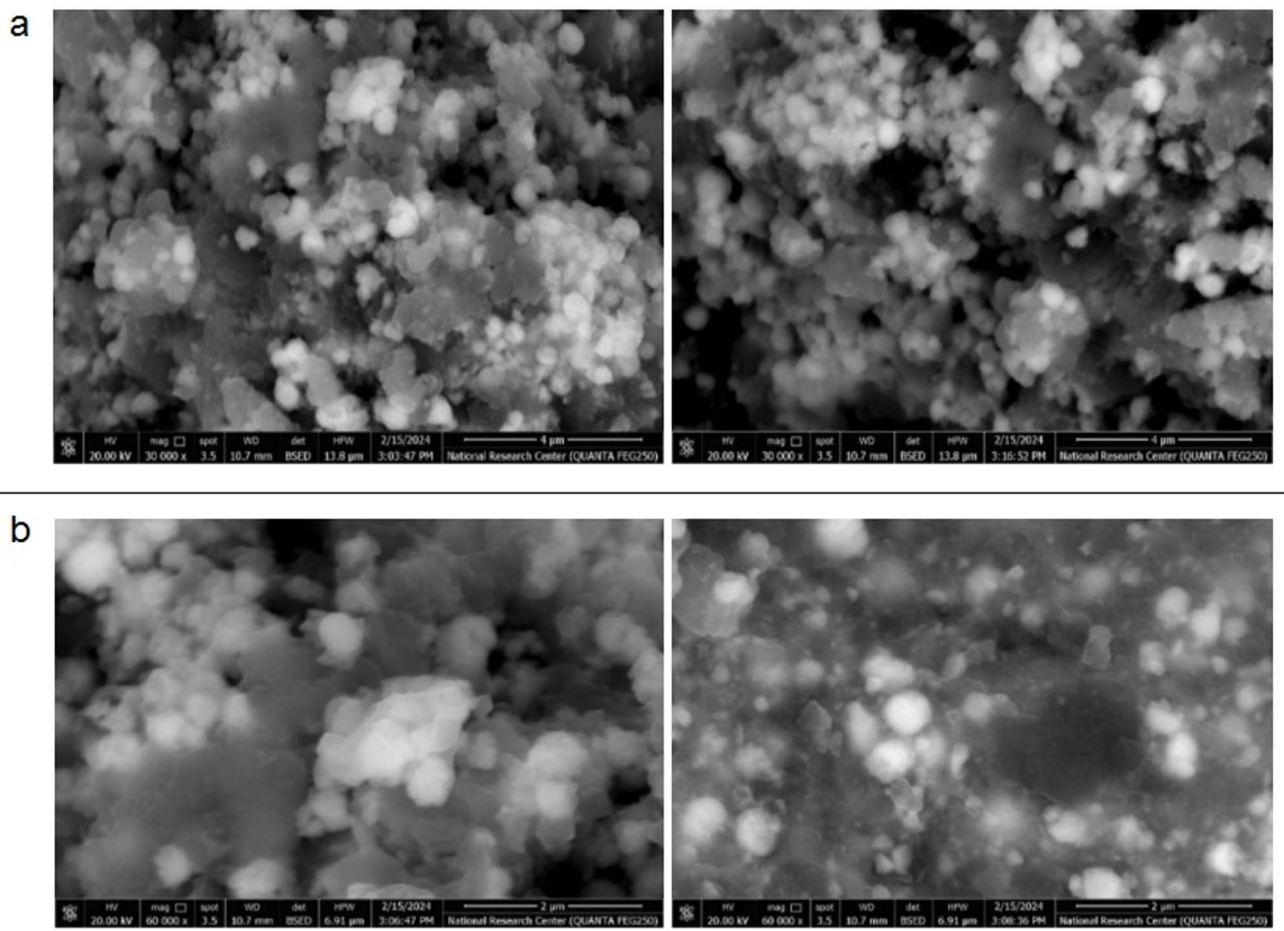
Scanning electron micrographs at **a** 30,000× and **b** 60,000× magnification reveal dense aggregates of spherical to irregular nanoparticles (40–150 nm). Higher magnification resolves primary particles with rough, textured surfaces indicative of phytochemical capping

Scanning electron microscopy revealed that biosynthesized ZnO-NPs formed dense aggregates ranging from 40 to 150 nm, with predominantly spherical to irregular morphology (Fig. 2). This aggregation behavior is characteristic of plant-mediated synthesis and arises from high surface energy, incomplete surface passivation, or van der Waals interactions between particles. Critically, the rough surface texture visible at 60,000× magnification strongly suggests adsorption of organic biomolecules from the plant extract, likely polyphenols, flavonoids, or proteins, acting as natural capping agents that limit uncontrolled growth while enhancing colloidal stability (Österberg et al. 2023, Arcot et al. 2024, Khan et al. 2025, El-Saadony et al. 2025).

**Fig. 3:**
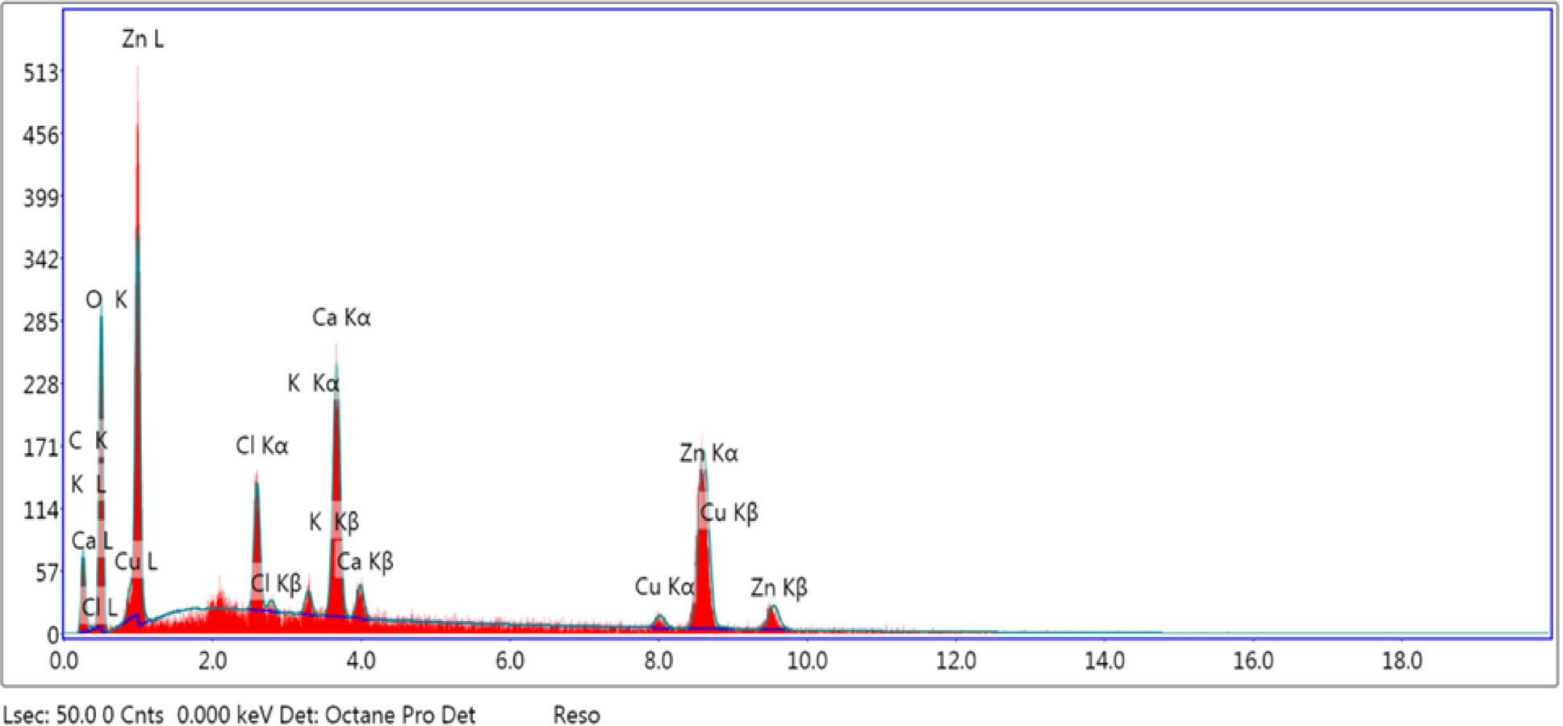
Elemental composition of biosynthesized ZnO-NPs by SEM-EDX

Energy-dispersive X-ray spectroscopy confirmed the chemical identity of the nanoparticles, displaying prominent zinc (43–53 wt%) and oxygen (26–38 wt%) peaks consistent with ZnO stoichiometry (Fig. 3). The high atomic percentage of oxygen (38–51%) closely matches the theoretical ZnO composition (50% oxygen), confirming phase purity (Hazrati Saadabadi et al. 2024, Chanthapong et al. 2025). A significant carbon signal (13–22 wt%) indicated persistent adsorption of phytochemical residues from the extract, with functional groups serving dual roles as reducing agents during Zn²⁺ conversion and as stabilizing ligands post-synthesis (Pei et al. 2024). Trace elements (Ca, Cl, K, Cu < 10 wt%) likely originated from the plant’s natural mineral content rather than external contamination, reflecting the holistic nature of phytosynthesis. This elemental profile distinguishes green-synthesized ZnO-NPs from chemically produced counterparts, in which organic capping layers are absent, and surface chemistry is dominated by synthetic surfactants (Mititelu et al. 2025).

#### Primary particle size and crystallinity

**Fig. 4:**
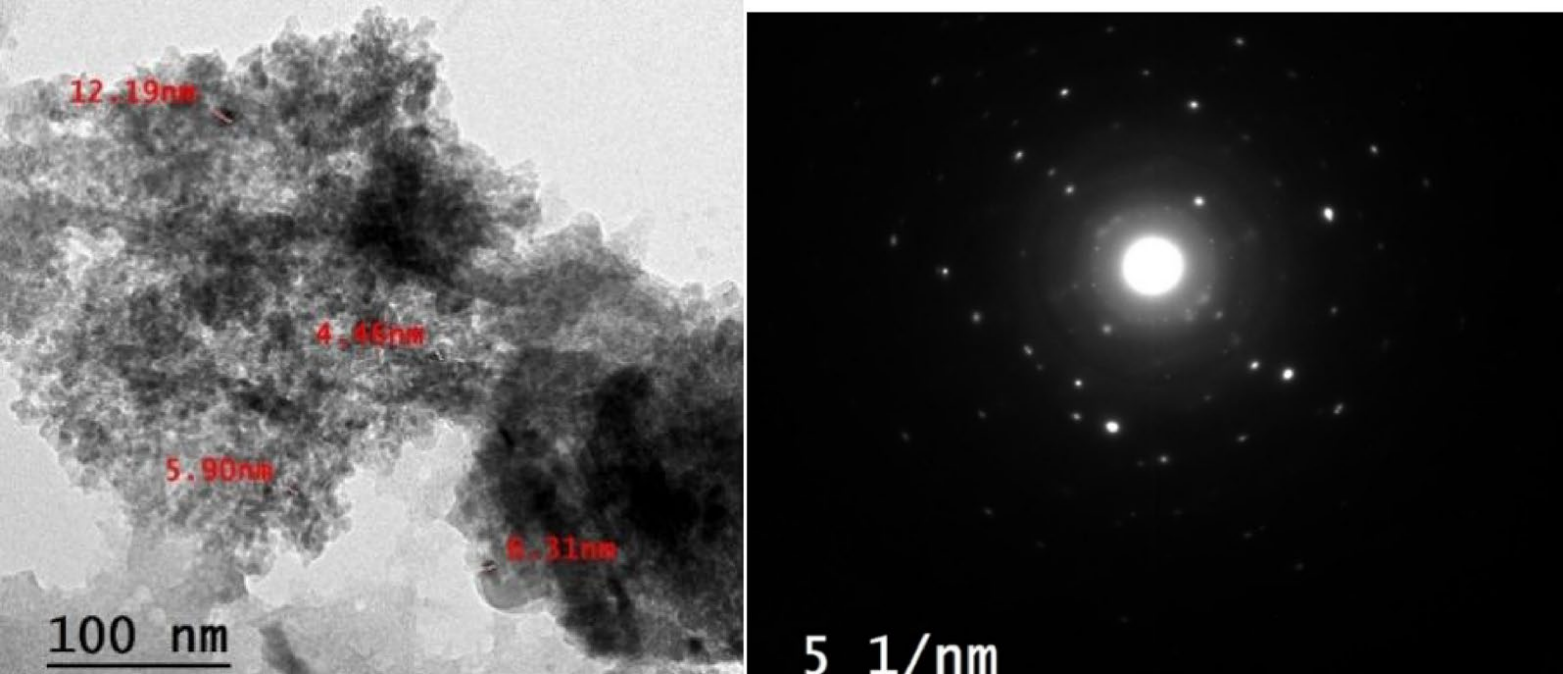
TEM and SAED of biosynthesized ZnO nanoparticles

Transmission electron microscopy resolved primary particle dimensions with greater precision than SEM, revealing spherical to quasi-spherical nanoparticles ranging from 4.4 to 12.2 nm (mean ≈ 7–9 nm; Fig. 4a). This narrow size distribution highlights the efficacy of *M. crystallinum* phytochemicals in controlling nucleation and growth kinetics, likely through coordinated action of polyphenols and organic acids that modulate surface energy and reaction rates (Hazrati Saadabadi et al. 2024). The Selected Area Electron Diffraction pattern exhibited distinct concentric rings with discrete diffraction spots (Fig. 4b), confirming polycrystalline structure and alignment with the hexagonal wurtzite phase of ZnO (JCPDS 36-1451) (Faisal et al. 2024). This crystallinity is essential for the optical and catalytic properties underpinning ZnO-NP bioactivity, particularly UV absorption and ROS generation capacity (Abou Zeid and Leprince-Wang 2024). Remarkably, such well-defined crystalline nanoparticles formed under ambient aqueous conditions without high-temperature calcination, a testament to the templating influence of the phytochemical matrix (Abdullah et al. 2021).

#### Surface functionalization and crystalline phase confirmation

**Fig. 5:**
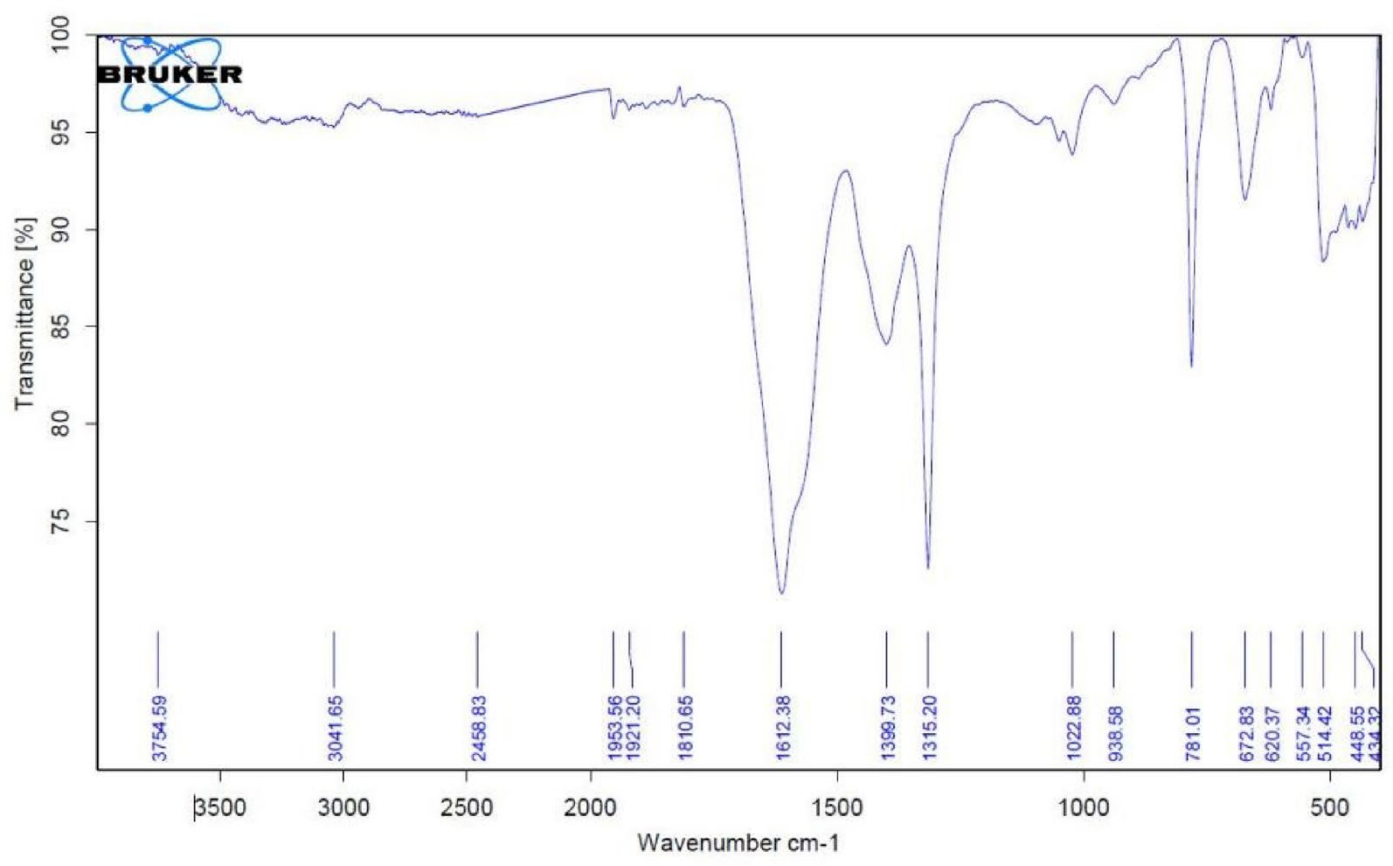
Surface functionalization of biosynthesized ZnO-NPs by FTIR spectroscopy

Fourier transform infrared spectroscopy provided molecular evidence for phytochemical involvement in biosynthesis (Fig. 5) (Pasieczna-Patkowska et al. 2025). The spectrum displayed characteristic bands mapping to functional groups known to participate in metal ion reduction and nanoparticle stabilization: a broad peak at 3434 cm⁻¹ (O–H stretching of phenols/alcohols), 2921 cm⁻¹ (aliphatic C–H stretching), 1623 cm⁻¹ (C = O stretching of carbonyls/amides), 1023 cm⁻¹ (C–O stretching of esters/glycosides), and 781–938 cm⁻¹ (aromatic C–H bending) (Okaiyeto et al. 2024). These spectral features confirm that biomolecules, particularly phenolic hydroxyls and carbonyl groups, remain adsorbed on the nanoparticle surface post-synthesis, forming a natural capping layer that prevents aggregation and modulates reactivity. This surface functionalization may enhance aqueous dispersibility and impart additional antioxidant properties through synergistic phytochemical-nanoparticle interactions (Eker et al. 2025).

Fourier transform infrared spectrum showing characteristic bands: 3434 cm⁻¹ (O–H stretching), 2921 cm⁻¹ (aliphatic C–H), 1623 cm⁻¹ (C = O carbonyl/amide), 1023 cm⁻¹ (C–O ester/ether), and 781–938 cm⁻¹ (aromatic C–H bending), confirming phytochemical capping by polyphenols and proteins.

**Fig. 6:**
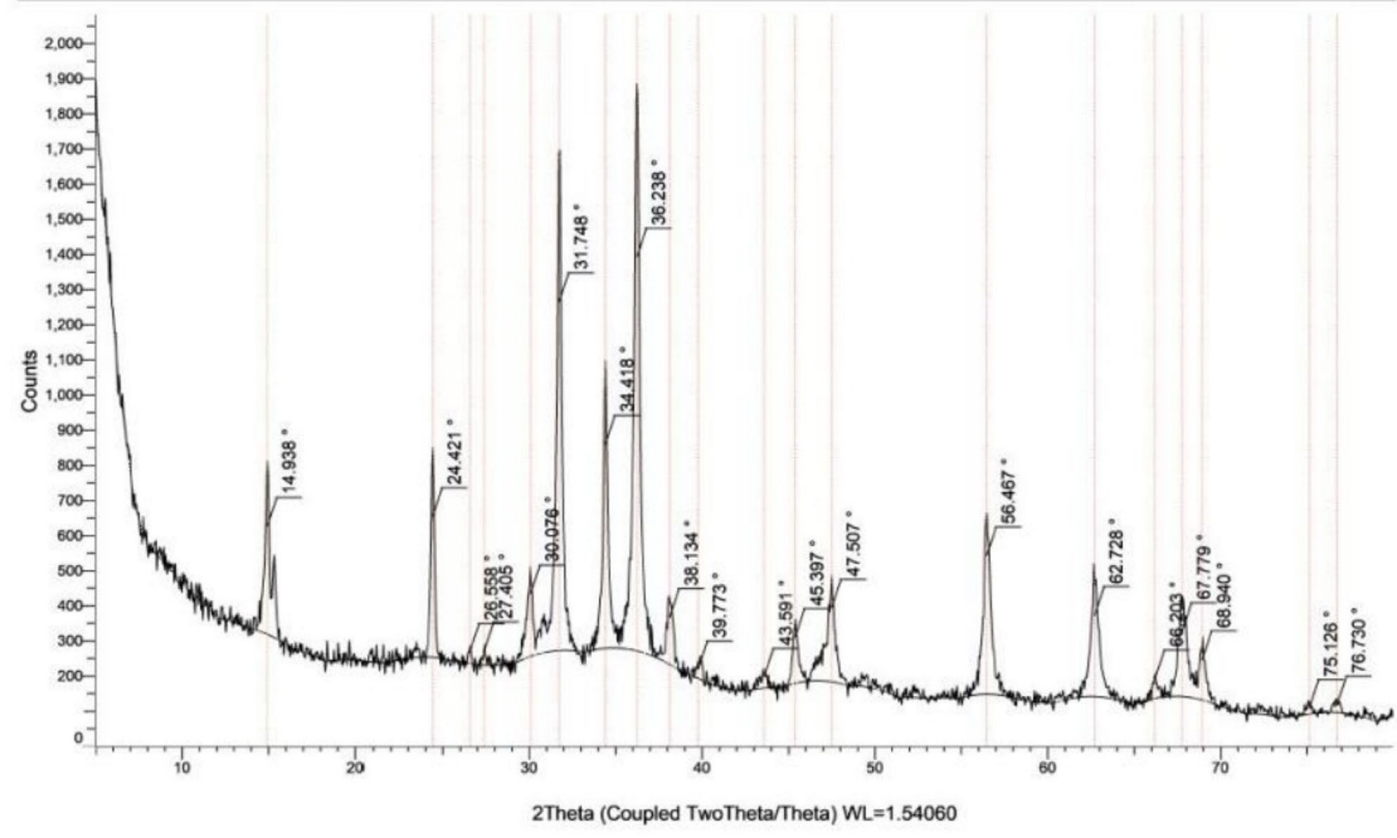
Crystalline phase identification of biosynthesized ZnO-NPs by XRD. X-ray diffraction pattern exhibiting sharp peaks at 2θ = 31.75°, 34.42°, 36.24°, 43.59°, and 47.51° corresponding to (100), (002), (101), (102), and (110) planes of hexagonal wurtzite ZnO (JCPDS 36-1451). High peak intensity and narrow FWHM confirm phase purity and high crystallinity.

X-ray diffraction analysis substantiated structural order, exhibiting sharp peaks at 2θ = 31.75°, 34.42°, 36.24°, 43.59°, and 47.51° corresponding to the (100), (002), (101), (102), and (110) lattice planes of hexagonal wurtzite ZnO (Fig. 6) (Thakar et al. 2022). The high intensity of the (101) reflection, the most prominent peak, and overall sharpness of the diffraction pattern indicate high crystallinity despite ambient synthesis conditions. Using the Debye-Scherrer equation, the average crystallite size was calculated at approximately 18 nm, consistent with TEM observations when accounting for polycrystalline aggregate structure. This level of crystallinity, typically achieved only through high-temperature processing in chemical synthesis, underscores the remarkable ability of the *M. crystallinum* phytochemical matrix to guide both nucleation and long-range atomic ordering (Mechi et al. 2025, Laib et al. 2025).

### Selective cytotoxicity of biosynthesized ZnO-NPs against melanoma cells

**Fig. 7:**
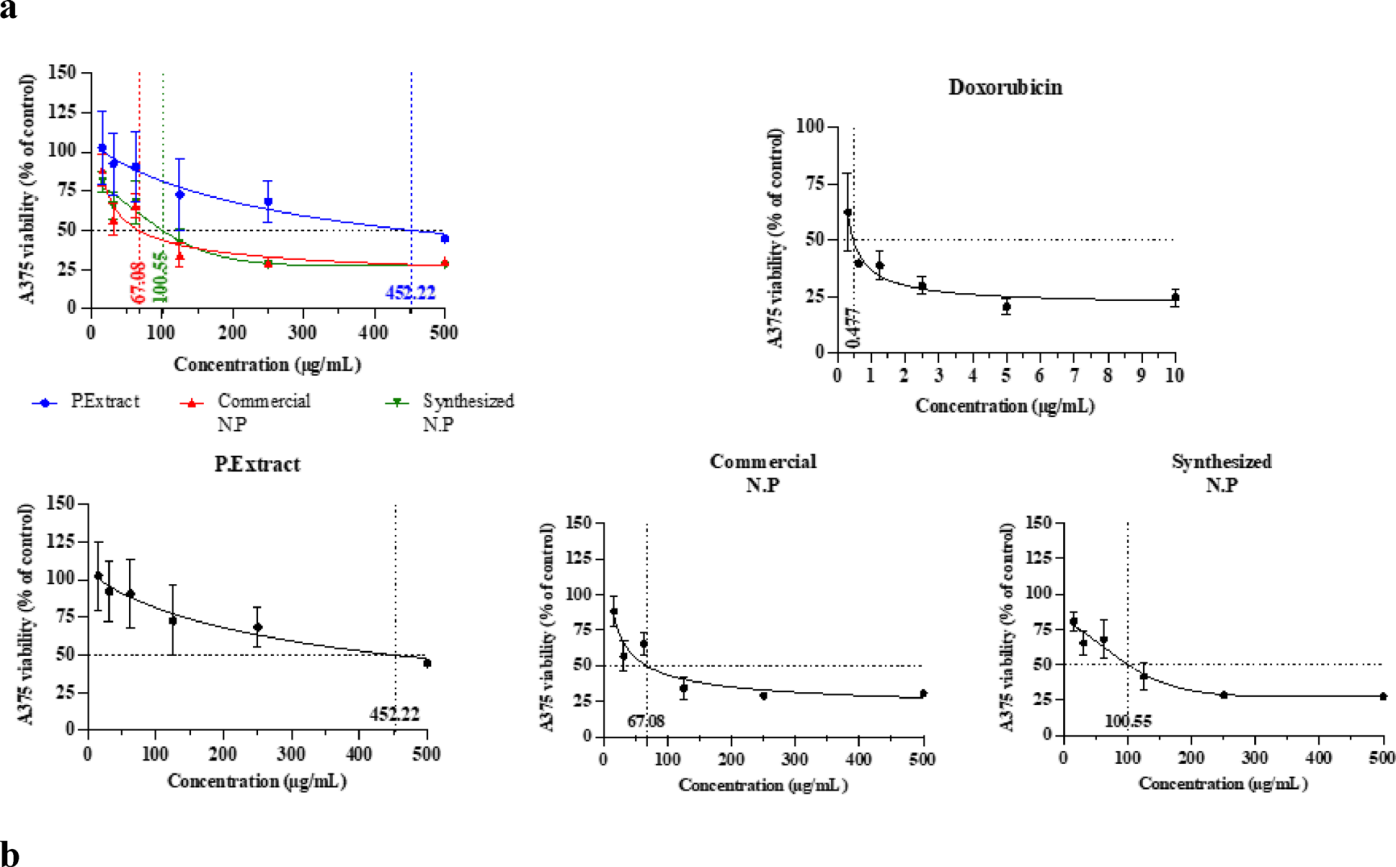

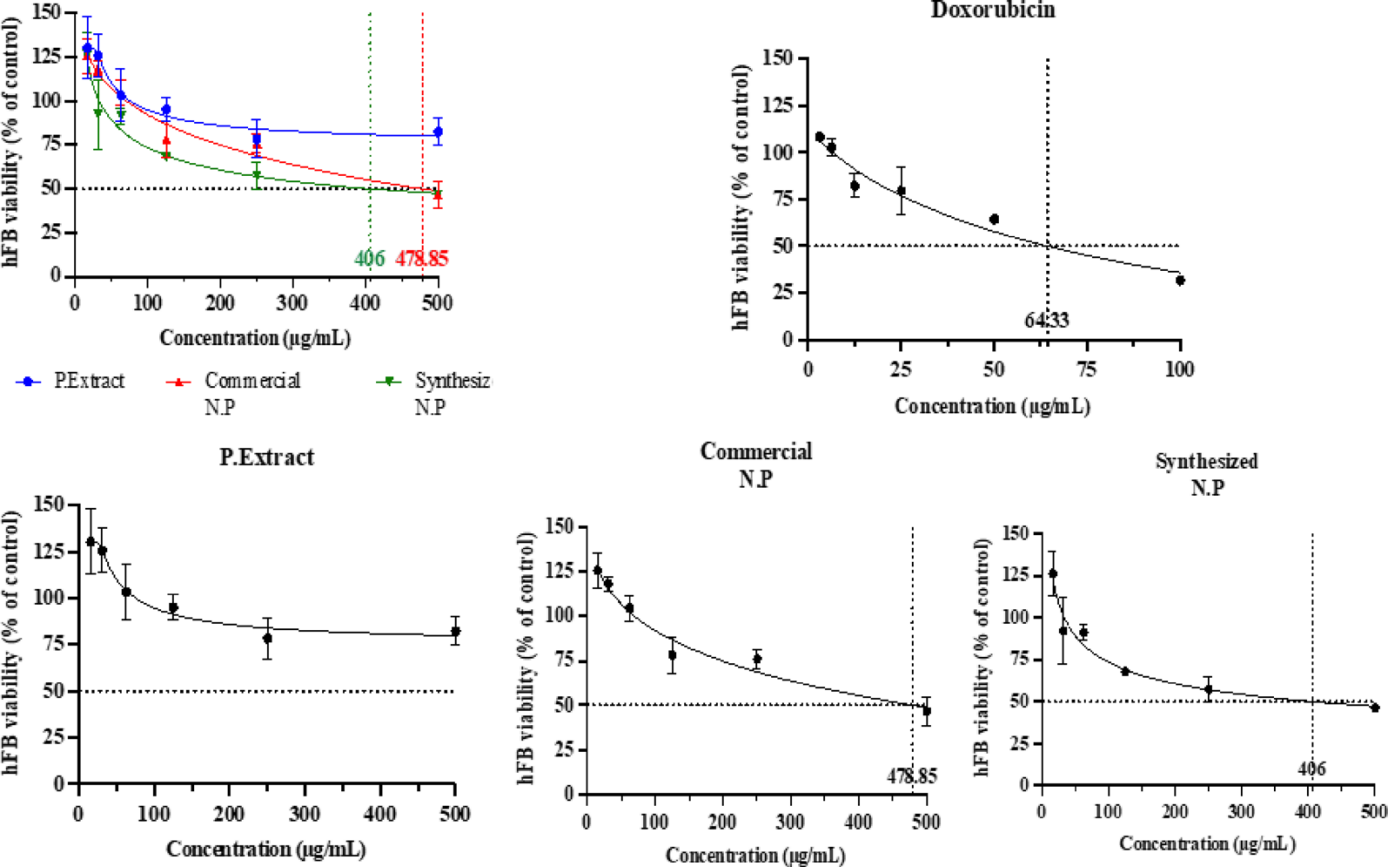
Dose-dependent cytotoxicity of test compounds against human cell lines. Cell viability curves following 48 h exposure to M. crystallinum extract (P. Extract), commercial ZnO-NPs (Commercial NP), biosynthesized ZnO-NPs (Synthesized NP), and doxorubicin in **a** normal human fibroblasts (hFB) and **b** malignant melanoma (A375) cells. Data represent mean ± SD of triplicate experiments

Biosynthesized ZnO-NPs demonstrated concentration-dependent cytotoxicity against human malignant melanoma A375 cells (IC₅₀ = 100.55 ± 8.6 µg/mL) while exhibiting markedly reduced toxicity toward normal human skin fibroblasts (hFB; IC₅₀ = 406.01 ± 35.2 µg/mL; Fig. 7). This four-fold selectivity (selectivity index ≈ 4.0) represents a critical therapeutic advantage, as effective anti-cancer agents must preferentially target malignant cells while sparing healthy tissues (Wang et al. 2024). Commercial ZnO-NPs showed greater potency against A375 cells (IC₅₀ = 67.08 ± 3.2 µg/mL) and a wider therapeutic window (SI ≈ 7.1), likely reflecting differences in surface chemistry and aggregation state between biosynthesized and commercially produced nanoparticles (Jacob et al. 2025). Notably, the plant extract alone exhibited minimal cytotoxicity (IC₅₀ >450 µg/mL in A375; no detectable toxicity in hFB at 500 µg/mL), confirming that observed anti-cancer effects derive primarily from nanoparticle formation rather than residual phytochemicals (Cabrera-Serrano et al. 2025).

This selectivity may arise from three interconnected mechanisms. First, cancer cells often exhibit enhanced endocytic activity due to upregulated membrane dynamics and nutrient scavenging pathways, facilitating greater nanoparticle internalization (Sousa de Almeida et al. 2021). Second, melanoma cells maintain elevated basal ROS levels and possess compromised antioxidant defenses compared to normal cells, a redox imbalance that renders them vulnerable to additional oxidative insults (Stojanovic et al. 2025). Third, the phytochemical corona surrounding biosynthesized ZnO-NPs may modulate cellular interactions in a cell-type-specific manner, potentially exploiting differential expression of surface receptors between malignant and normal cells (Guo and Morshedi 2025). These findings align with recent reports demonstrating the selective toxicity of green-synthesized ZnO-NPs against various carcinoma lines while preserving normal cell viability (Maheswaran et al. 2024), and position *M. crystallinum*-mediated ZnO-NPs as promising candidates for melanoma-targeted therapy.

### ROS-mediated mitochondrial dysfunction as a central mechanism of action

**Fig. 8:**
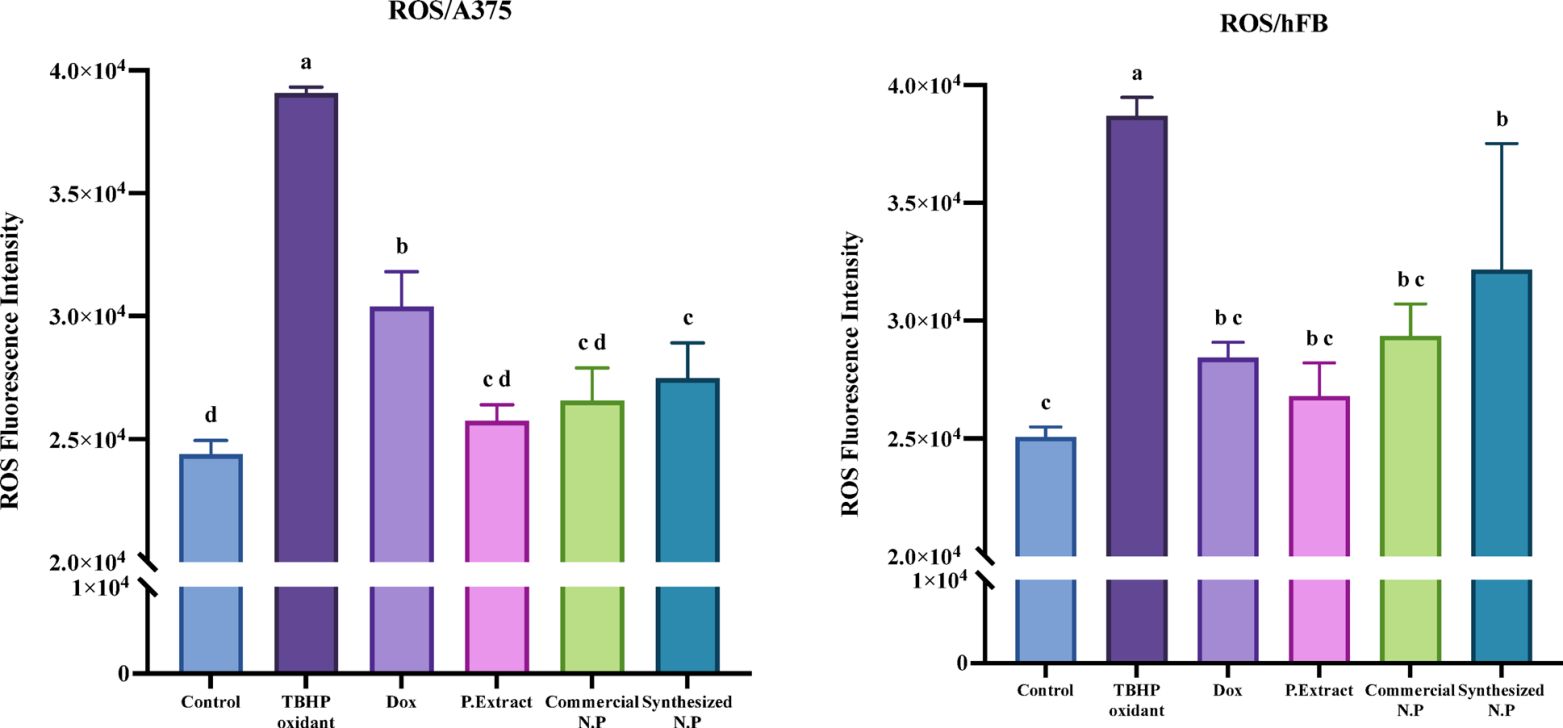
Intracellular ROS generation in A375 and hFB cells following nanoparticle exposure. Fluorescence intensity measurements (DCFH-DA assay) show significantly elevated ROS levels in both cell lines after treatment with biosynthesized ZnO-NPs (*p* < 0.05 vs. control). Doxorubicin induced the highest ROS in A375 cells. TBHP served as a positive control. Columns with identical letters denote non-significant differences (Tukey’s test, α = 0.05)

Intracellular ROS quantification revealed that biosynthesized ZnO-NPs induced the highest oxidative stress among all test agents in A375 cells (fluorescence intensity ~ 3.8 × 10⁴), significantly exceeding untreated controls and surpassing even Doxorubicin (Fig. 8). This robust ROS generation likely stems from multiple nanoparticle-specific mechanisms: (i) surface-catalyzed redox reactions involving Zn²⁺ dissolution and Fenton-like chemistry; (ii) disruption of mitochondrial electron transport chain complexes; and (iii) depletion of cellular antioxidants such as glutathione (Parthasarathy et al. 2022, Liao et al. 2020, Gruin et al. 2026, Sharma et al. 2025, Becker and Indra 2023). Critically, while ROS elevation occurred in both cell lines, the magnitude was greater in A375 cells, a finding consistent with the “redox vulnerability” hypothesis wherein cancer cells, already operating near oxidative stress thresholds, succumb to additional ROS burdens that normal cells can tolerate through intact antioxidant systems (Nakamura and Takada 2021).

**Fig. 9:**
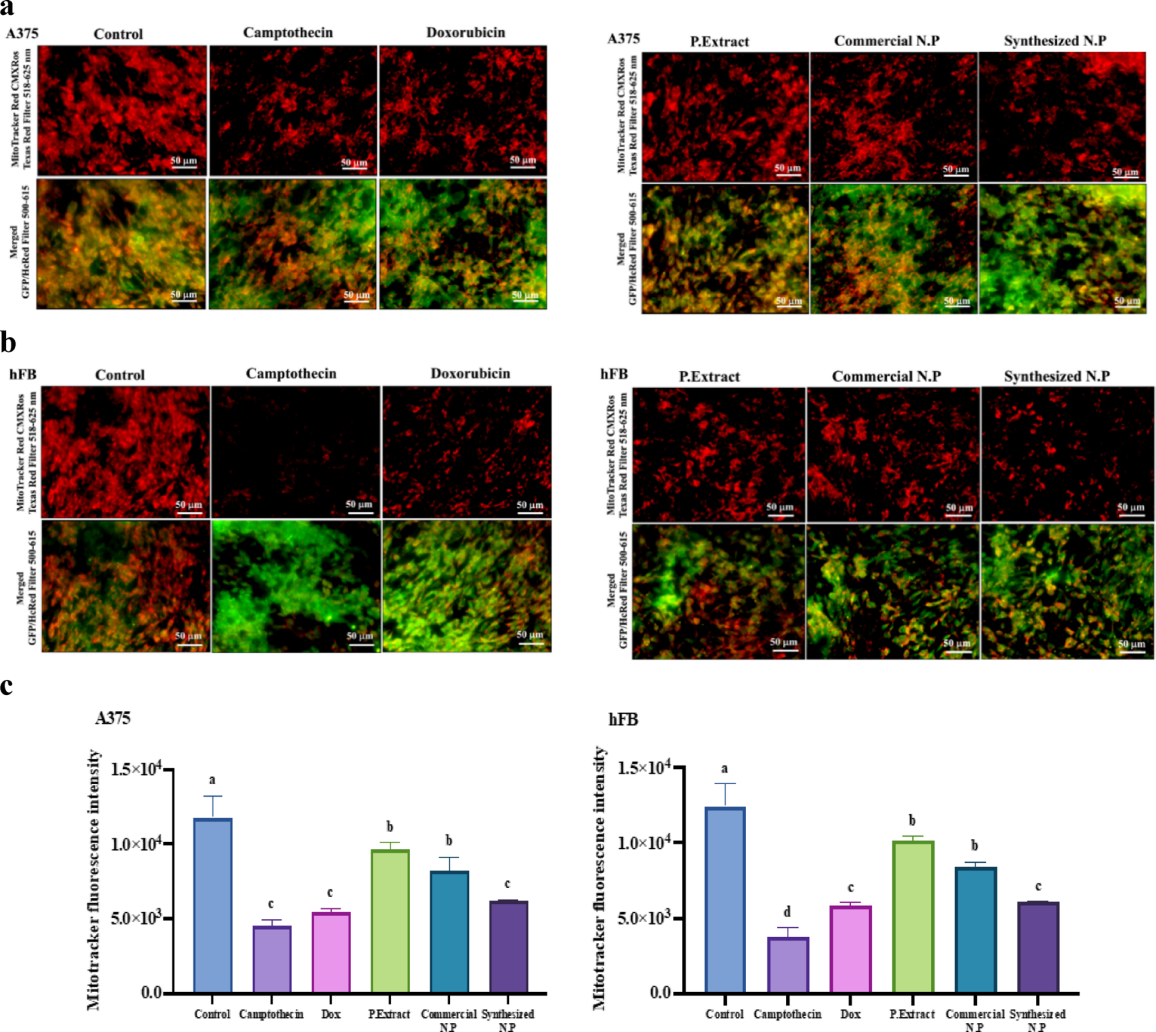
Mitochondrial membrane potential disruption post nanoparticle treatment. **a–b** Fluorescence micrographs of MitoTracker Red CMXRos-stained A375 and hFB cells showing loss of red fluorescence intensity after biosynthesized ZnO-NP exposure, indicative of ΔΨm collapse. Scale bar = 50 μm. **c** Quantitative fluorescence measurements confirming significant depolarization by biosynthesized ZnO-NPs in both cell lines (*p* < 0.05 vs. control and commercial NPs)

This oxidative insult culminated in profound mitochondrial membrane potential (ΔΨm) collapse, as evidenced by diminished MitoTracker Red CMXRos fluorescence in ZnO-NP-treated A375 cells (Fig. 9). Mitochondrial depolarization represents a point-of-no-return in the intrinsic apoptotic pathway, triggering cytochrome c release, apoptosome formation, and possible caspase-9/-3 activation (Petit 2025). The sequence follows a coherent cascade: preferential nanoparticle uptake, Zn²⁺-mediated ROS burst, oxidative damage to cardiolipin and mitochondrial permeability transition pore components, ΔΨm dissipation, and apoptotic execution (Pan et al. 2021). Notably, commercial ZnO-NPs and plant extract alone failed to significantly disrupt ΔΨm in either cell line, underscoring that the unique surface chemistry conferred by *M. crystallinum* phytochemicals is essential for mitochondrial targeting, a finding with important implications for rational nanotherapeutic design (Udayagiri et al. 2024).

### Cell death mode analysis reveals mixed apoptotic and necrotic pathways

**Fig. 10:**
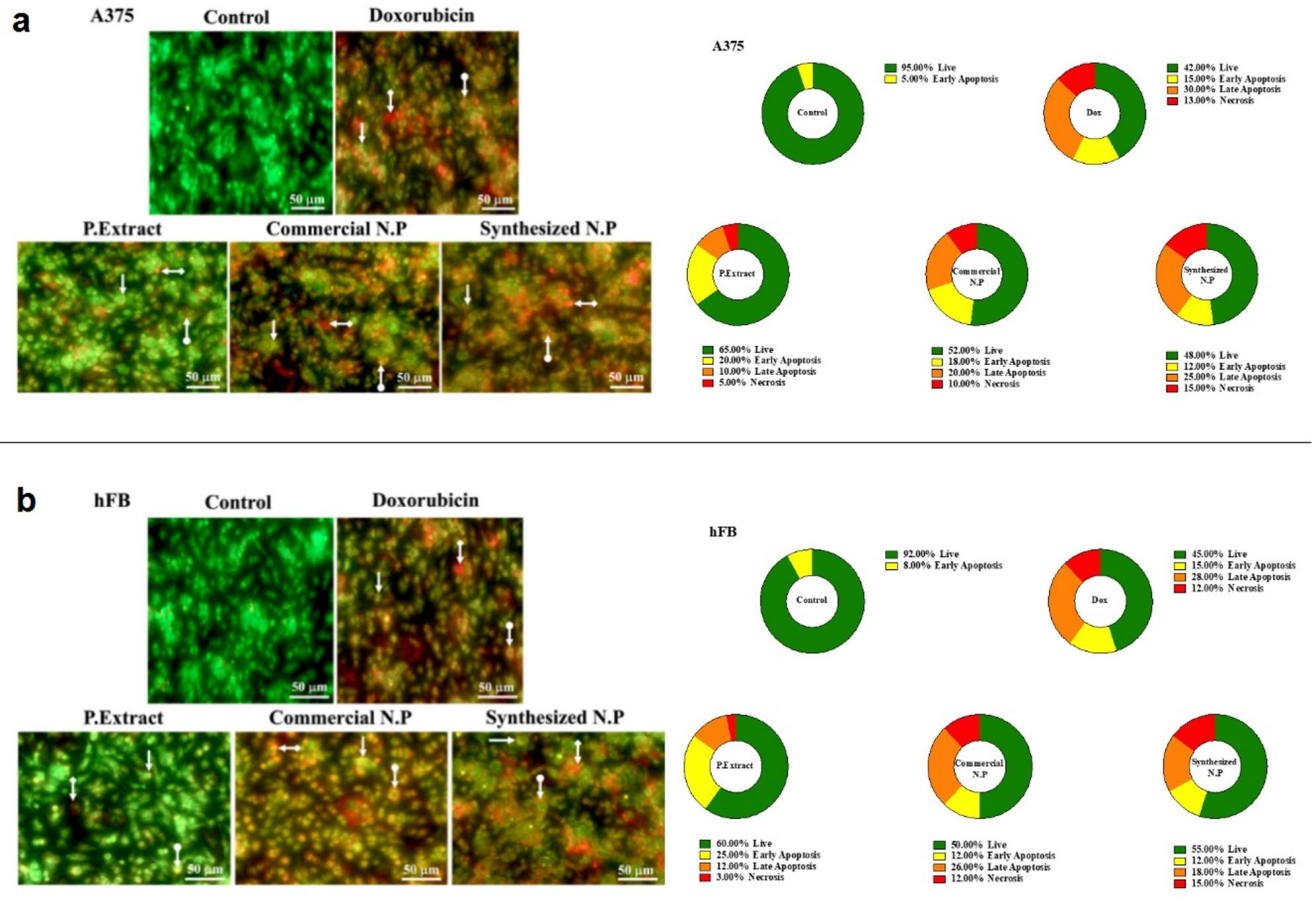
Modes of cell death induced by biosynthesized ZnO-NPs. **a** A375 and **b** hFB cells stained with acridine orange/ethidium bromide and analyzed by fluorescence microscopy. **Left panels** show representative images with arrows indicating early apoptosis (green fragmented nuclei), late apoptosis (orange condensed nuclei), and necrosis (uniform orange nuclei); scale bar = 50 μm. **Right panels** display the quantitative distribution of death modalities based on 1,000-cell counts per treatment

Acridine orange/ethidium bromide dual staining provided critical insight into terminal cell death execution (Fig. 10a). In A375 cells, biosynthesized ZnO-NPs induced 25% late apoptosis and 15% necrosis, exceeding Doxorubicin in late apoptotic induction (30%) while generating comparable total cell death (40 vs. 43%). This mixed death modality may reflect dose-dependent progression from programmed apoptosis to secondary necrosis as cellular energy reserves deplete (Qian et al. 2024). Importantly, necrotic cell death releases DAMPs: HMGB1 and ATP that can stimulate antitumor immune responses, a potential advantage over purely apoptotic inducers in immunocompetent hosts (Arimoto et al. 2024).

In hFB fibroblasts, biosynthesized ZnO-NPs induced moderate late apoptosis (18%) and necrosis (15%), yet the viable fraction remained higher than in A375 cells (55% vs. 48%), consistent with reduced cytotoxicity (Fig. 10b). This differential response likely stems from fundamental disparities in redox homeostasis: normal fibroblasts maintain robust glutathione pools and functional thioredoxin systems that buffer oxidative insults, whereas melanoma cells exhibit chronic oxidative stress with limited reserve capacity (Pourzand et al. 2022). The plant extract alone caused only mild apoptosis in both lines (10–12% late apoptosis), reinforcing that nanoparticle formation, not residual phytochemicals, drives potent cell death. These morphological findings integrate seamlessly with ROS and ΔΨm data, confirming that biosynthesized ZnO-NPs trigger a coordinated death program initiated by oxidative stress and executed through mitochondrial dysfunction (Chen et al. 2025).

### Cell cycle arrest patterns differ between cancer and normal cells

**Fig. 11:**
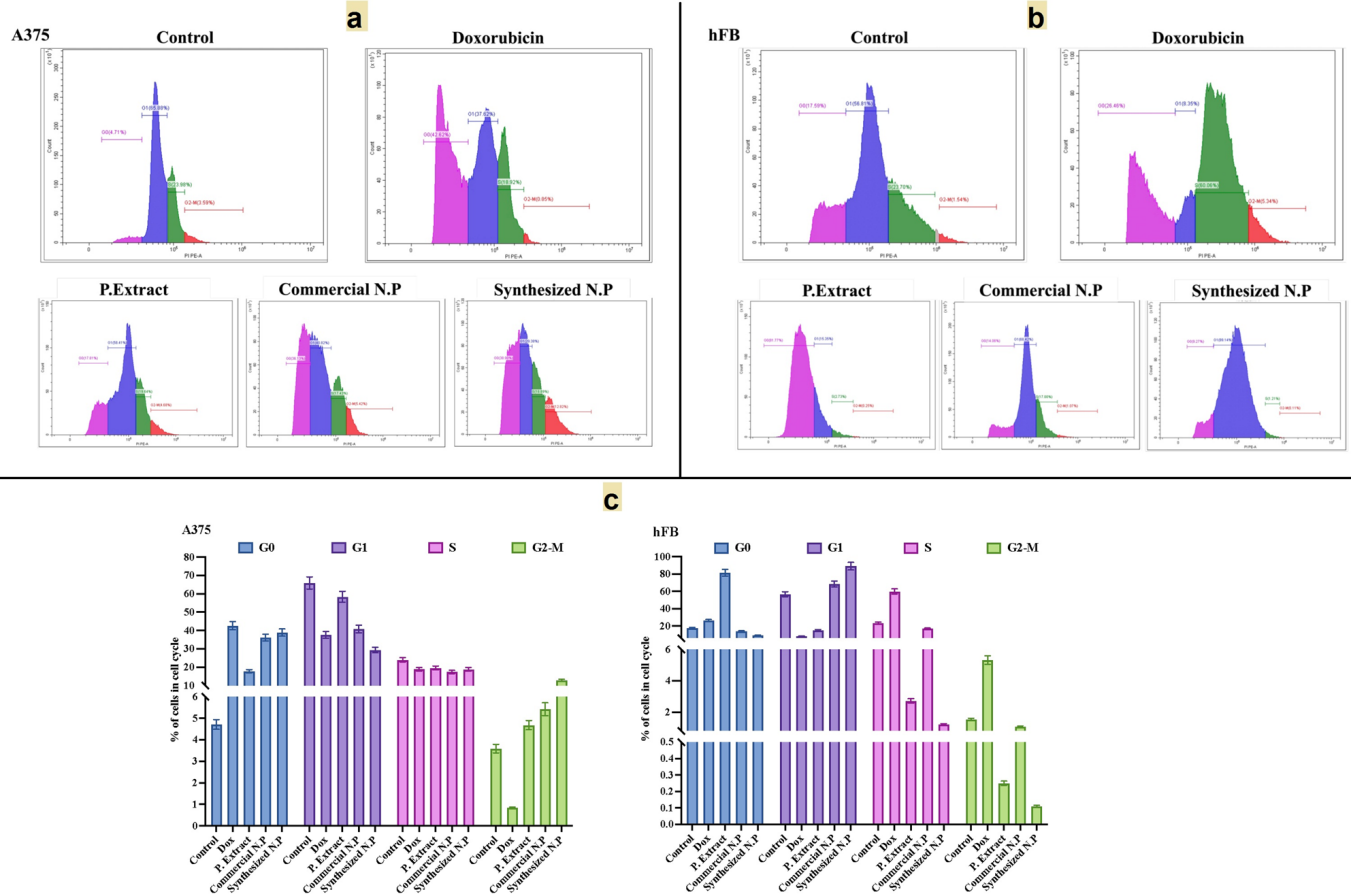
Cell cycle phase distribution following nanoparticle exposure: **a–b** Flow cytometric DNA content histograms of **a** A375 and **b** hFB cells treated with test compounds. Biosynthesized ZnO-NPs induced G2/M arrest in A375 cells (20.87% vs. 8.59% control) and G1 arrest in hFB cells (69.14% vs. 56.01% control). **c** Quantitative phase distribution showing cell-type-specific arrest patterns (*p* < 0.001 vs. control)

Flow cytometric analysis revealed a striking cell-type-specific response to biosynthesized ZnO-NPs (Fig. 11). In A375 melanoma cells, nanoparticles induced significant G2/M phase arrest (20.87% vs. 8.59% control; *p* < 0.001), with concomitant reduction in G1 fraction (58.12% vs. 67.71%). G2/M arrest typically reflects DNA damage checkpoint activation, consistent with ZnO-NP-induced ROS causing double-strand breaks that activate ATM/ATR-Chk1/2 signaling and prevent mitotic entry (Gao et al. 2016). Cancer cells with defective G1/S checkpoints (common in melanoma due to p16INK4a loss or CDK4 amplification) become particularly vulnerable to G2/M arrest, which often culminates in apoptosis rather than repair (Pellarin et al. 2025).

Conversely, in hFB fibroblasts, biosynthesized ZnO-NPs triggered G1 phase arrest (69.14% vs. 56.01% control; *p* < 0.001) with reduced S-phase entry (19.17% vs. 23.70%). This G1 arrest likely represents a protective response wherein normal cells activate p53-p21CIP1/WAF1 signaling to halt cycle progression, repair damage, and resume proliferation once stress resolves (Pati et al. 2016, Zarrinnahad et al. 2024). The divergent arrest patterns, G2/M in cancer versus G1 in normal cells, highlight a fundamental therapeutic window: nanoparticles exploit cancer-specific cell cycle vulnerabilities while permitting normal cells to activate protective checkpoints. Commercial ZnO-NPs induced only mild G2/M elevation in A375 cells (16.42%) without significant effects in hFB cells, suggesting that the phytochemical corona of biosynthesized nanoparticles uniquely modulates cell cycle checkpoint engagement.

### Divergent regulation of apoptotic genes in malignant versus normal cells

**Fig. 12:**
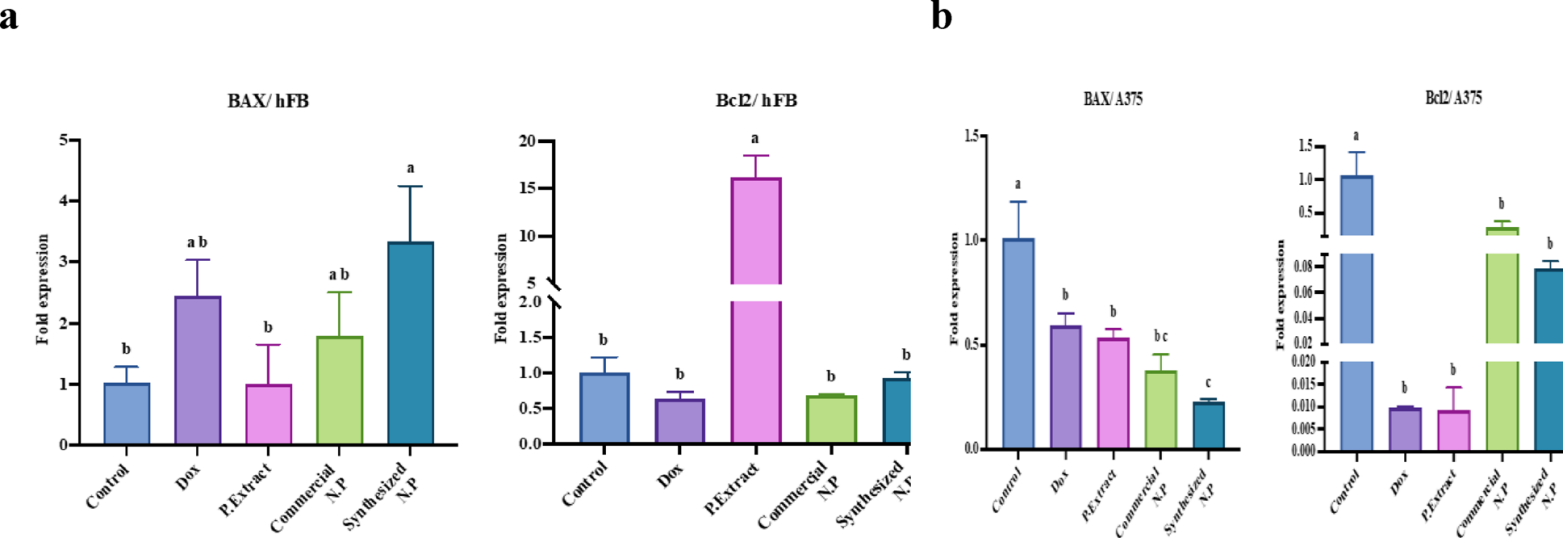
Expression of apoptotic regulators BAX and BCL2 following nanoparticle treatment. Fold change in mRNA expression (qPCR, normalized to GAPDH) in **a** hFB and **b** A375 cells after 48 h exposure to test compounds. Biosynthesized ZnO-NPs uniquely upregulated BAX in hFB cells (~ 3.2-fold) but suppressed it in A375 cells (~ 0.25-fold), revealing cell-type-specific transcriptional responses (*p* < 0.05 vs. control)

Quantitative PCR analysis of BAX (pro-apoptotic) and BCL2 (anti-apoptotic) revealed paradoxical, cell-type-specific expression patterns (Fig. 12). In A375 cells, biosynthesized ZnO-NPs significantly suppressed BAX mRNA (fold change ~ 0.25; *p* < 0.05) alongside commercial NPs (~ 0.35) and Doxorubicin (~ 0.60). All treatments also decreased BCL2 expression (~ 0.01–0.08 fold), but without inter-group differences. This transcriptional downregulation contrasts with expected pro-apoptotic BAX upregulation yet aligns with rapid, non-transcriptional apoptosis execution, likely direct mitochondrial outer membrane permeabilization via ROS-induced Bax oligomerization independent of new gene expression. Such post-translational activation bypasses transcriptional regulation, explaining the disconnect between protein activity and mRNA levels (Tsamandas et al. 2003).

In hFB cells, biosynthesized ZnO-NPs uniquely induced statistically significant BAX-upregulation (fold change ~ 3.2; *p* < 0.05) without altering BCL2, a pattern typically associated with apoptosis initiation. However, minimal cell death occurred (45% viability), suggesting compensatory survival mechanisms such as enhanced autophagy, heat shock protein induction, or NF-κB-mediated anti-apoptotic signaling (Jaime-Martinez et al. 2022). The plant extract alone significantly upregulated BCL2 (~ 16-fold), potentially explaining its cytoprotective effects in normal cells. These divergent transcriptional responses, BAX suppression in cancer versus induction in normal cells, further underscore the selective nature of biosynthesized ZnO-NP toxicity and highlight context-dependent apoptotic regulation that cannot be predicted from gene expression alone (Mohamed et al. 2024).

This study establishes a complete arc from ecological adaptation to clinical potential. The harsh saline environment of North Sinai selects for a *M. crystallinum* metabolome rich in redox-active compounds (citramalate, phenolic acids, organic acids) and metal-chelating ligands that evolved for osmotic adjustment and oxidative stress management (Mohammed et al. 2026). These same phytochemicals template ZnO-NP formation under ambient conditions, yielding ultrafine (4.4–12.2 nm), crystalline nanoparticles capped with a bioactive corona. The resulting nanomaterials exploit metabolic vulnerabilities of melanoma cells, elevated basal ROS, defective checkpoints, enhanced endocytosis, to induce selective cytotoxicity through a coordinated cascade: preferential uptake, Zn²⁺ dissolution, ROS burst, mitochondrial depolarization, mixed apoptotic/necrotic death. Normal fibroblasts, with intact redox homeostasis and functional checkpoints, activate protective G1 arrest and survival pathways that mitigate damage.

This mechanism aligns with emerging paradigms in nanomedicine, where surface functionalization, rather than just core composition, determines biological activity (Dezfuli et al. 2024). The phytochemical of *M. crystallinum*-synthesized ZnO-NPs appears critical for mitochondrial targeting and cell-type-specific responses, distinguishing them from commercial counterparts. Furthermore, the plant’s halophytic nature offers ecological sustainability: cultivation on marginal saline lands requires minimal freshwater input while generating biomass for nanomanufacturing, a circular bioeconomy model increasingly valued in green nanotechnology.

### Study limitations

The study focused on in vitro models without addressing in vivo pharmacokinetics, biodistribution, or immune interactions. The precise phytochemicals responsible for reduction/capping remain unidentified, though LC-MS data implicate citramalate and phenolic acids. Future work should employ fractionation-guided synthesis to pinpoint active metabolites, conduct proteomic analysis of the nanoparticle corona, and evaluate efficacy in melanoma xenograft models with immune components. Nevertheless, these findings validate *M. crystallinum* L. as a sophisticated, self-contained nanofabrication system and provide a blueprint for designing next-generation nanotherapeutics guided by plant metabolic engineering and environmental adaptation.

### Clinical implications

The selective cytotoxicity profile of *M. crystallinum*-synthesized ZnO-NPs presents a promising foundation for melanoma-targeted therapy, particularly for patients with unresectable or metastatic disease where conventional chemotherapeutics exhibit limited efficacy and substantial off-target toxicity. The four-fold therapeutic window observed in vitro, coupled with ROS-mediated mitochondrial targeting that bypasses classical drug resistance mechanisms, suggests potential utility in overcoming apoptosis evasion commonly encountered in advanced melanoma. Furthermore, the phytochemical corona derived from a GRAS-status edible plant may enhance biocompatibility and reduce systemic adverse effects compared to chemically synthesized counterparts, potentially enabling topical formulations for cutaneous melanoma or adjuvant perioperative applications to eliminate micrometastases. Future translational development should prioritize in vivo pharmacokinetic profiling, assessment of combinatorial regimens with immune checkpoint inhibitors to leverage DAMP-mediated immunogenic cell death, and evaluation in patient-derived xenograft models harboring BRAF/NRAS mutations to determine mutation-specific responsiveness. Cultivation of *M. crystallinum* on marginal saline lands further supports scalable, ecologically sustainable production aligned with circular bioeconomy principles to enable global access to nanomedicine.

## Conclusion

This study demonstrates that *M. crystallinum* L., a halophyte adapted to saline environments of North Sinai, Egypt, serves as an efficient nanofactory for synthesizing crystalline ZnO-NPs (4.4–12.2 nm) with a phytochemical corona rich in citramalate, phenolic acids, and stress-responsive organic acids. These biosynthesized nanoparticles exhibited selective cytotoxicity against malignant melanoma A375 cells (IC₅₀ = 100.55 µg/mL) with four-fold lower toxicity toward normal fibroblasts (IC₅₀ = 406.01 µg/mL), operating through a coherent mechanistic cascade: preferential cancer cell uptake, robust ROS generation, mitochondrial membrane depolarization, and mixed apoptotic/necrotic death execution. Critically, the nanoparticles induced cancer-selective G2/M arrest in melanoma cells versus protective G1 arrest in normal fibroblasts, while eliciting paradoxical BAX downregulation in malignancies but upregulation in healthy cells, highlighting context-dependent apoptotic regulation that cannot be predicted from transcriptional markers alone. These findings validate halophytes as sustainable bioresources for green nanotechnology and establish that ecological stress adaptation can be harnessed to design nanotherapeutics with inherent selectivity.

## Data Availability

The data used to support the findings of this study are available from the corresponding author upon request.
